# Network Pharmacology and Molecular Docking–Based Investigation: *Prunus mume* Against Colorectal Cancer via Silencing *RelA* Expression

**DOI:** 10.3389/fphar.2021.761980

**Published:** 2021-11-19

**Authors:** Minfeng Zhou, Jinxiao Li, Dan Luo, Haiming Zhang, Zhaomin Yu, Youlin Chen, Qiumeng Li, Fengxia Liang, Rui Chen

**Affiliations:** ^1^ Department of Integrated Traditional Chinese and Western Medicine, Union Hospital, Tongji Medical College, Huazhong University of Science and Technology, Wuhan, China; ^2^ Department of Respiratory Medicine, Wuhan First Hospital, Tongji Medical College, Huazhong University of Science and Technology, Wuhan, China; ^3^ Department of Oncology, Integrated Traditional Chinese and Western Medicine, The Central Hospital of Wuhan, Tongji Medical College, Huazhong University of Science and Technology, Wuhan, China; ^4^ Department of Oncology, Hubei Provincial Hospital of Integrated Chinese and Western Medicine, Wuhan, China; ^5^ School of Resources and Environment Science, Wuhan University, Wuhan, China; ^6^ Clinical College of Traditional Chinese Medicine, Hubei University of Chinese Medicine, Wuhan, China; ^7^ College of Acupuncture & Moxibustion and Orthopaedics, Hubei University of Chinese Medicine, Wuhan, China

**Keywords:** *Prunus mume*, colorectal cancer, network pharmacology, moleculardocking, RelA, apoptosis

## Abstract

Colorectal cancer (CRC) is one of the most pervasive cancers in the human disease spectrum worldwide, ranked the second most common cause of cancer death by the end of 2020. *Prunus mume* (*PM*) is an essential traditional Chinese medicine for the adjuvant treatment of solid tumors, including CRC. In the current study, we utilize means of network pharmacology, molecular docking, and multilayer experimental verification to research mechanism. The five bioactive compounds and a total of eight critical differentially expressed genes are screened out using the bioinformatics approaches of Cytoscape software, String database, Gene Ontology analysis, Kyoto Encyclopedia of Genes and Genomes pathways, and molecular docking. *RelA* has been proven to be highly expressed in CRC. Experiments *in vitro* have shown that kaempferol, the main active component of *PM*, dramatically inhibited the growth, migration, and invasion of CRC cells, and experiments *in vivo* have shown that *PM* effectively delays CRC formation and improves the survival cycle of mice. Further analysis shows that *PM* inhibits the CRC progression by down-regulating the expression level of *RelA*, *Bax*, *caspase 3*, *caspase 9*, and *EGFR* in CRC. *PM* and its extract are potentially effective therapeutics for the treatment of CRC via the *RelA*/nuclear factor κB signaling pathway.

## Highlights

1. Components of *Prunus mume* (*PM*) and differentially expressed genes (DEGs) of colorectal cancer (CRC) screening by intelligent network pharmacology and molecular docking.
2. Bioinformatics approaches of Cytoscape software, String database, Gene Ontology analysis, Kyoto Encyclopedia of Genes and Genomes pathways, and molecular docking applying to select the critical genes of *PM*-DEGs.
3. *RelA* proved to be highly expressed in CRC in human CRC tissue *in vitro* and *in vivo*.
4. First report on the comprehensive study to explore the molecular mechanism of *PM* in the treatment of CRC.

## Introduction

Colorectal cancer (CRC) without obvious symptoms expresses early the main symptoms of occult or overt rectal bleeding, anemia, change in defecation habits, or abdominal pain in an advanced period (Dekker et al., 2019). CRC had been ranked the second most common cause of cancer death in America by 2020. Morbidity and mortality rates were increasing recently by 1% and 1.3% per year among the population younger than 65 years (Siegel et al., 2020). As screening programs and colonoscopies increase, CRC morbidity and mortality are stabilized and decline in developed countries. Still, with progress in developing countries, the global incidence of CRC will continue to increase (Bray et al., 2018; Dekker et al., 2019). The CRC incidence continues to rise in countries with medium to high human development index (HDI) (Wong et al., 2021). Unreasonable diet structure, overweight, physical inactivity, smoking, ultraviolet radiation, and cancer-related infections are modifiable factors leading to the development of CRC (Islami et al., 2018). Endoscopic and surgical resection, preoperative chemotherapy, local metastatic ablation, palliative chemotherapy, local radiotherapy, targeted therapy, biologic therapy, and immunotherapy are current treatments for CRC treatment (Dekker et al., 2019). However, the risk of recurrence, metastasis, and severe side effects is inevitable. As adjunctive therapy, traditional Chinese herbal medicine (CHM) is expected to play a role in reducing toxicity and enhancing efficacy in the treatment of CRC.

Traditional Chinese medicine (TCM) is natural medicine, including plants, animals, and minerals. As most TCM are plants, TCM is also called CHM (Qi et al., 2020). CHM and their active components played a positive role in the treatment of multiple cancers (Wang et al., 2021), including combination therapy after first-line treatment for the non–small cell lung cancer (Wang et al., 2018), triple-negative breast cancer (Yang et al., 2021), liver cancer (Liu et al., 2018a), gastric cancer (Zhou et al., 2019), and CRC (Wang et al., 2021). As a representative herb vested in the homology of medicine and food, the safety of *Prunus mume* (*PM*) can be guaranteed. *PM* has the function of inhibiting a variety of inflammatory factors, anti-inflammation and antioxidation, repairing gastrointestinal tract, and regulating gastrointestinal movement and intestinal secretion (Xing et al., 2018). *PM* treated diarrhea and improved intestinal symptoms caused by lapatinib combined with capecitabine. *PM* plays anti-inflammatory effects by inhibiting MBP degradation and activating intracellular signals, including TLR4 and P38 MAPK, thus improving brain injury and becoming a potential drug for treating vascular dementia (Lee et al., 2015). Mumefural (MF), a component of *PM*, improved cognitive impairment in BCCAO rats by enhancing neurological function and inhibiting neuroinflammation (Bang et al., 2019). *PM* inhibits the proliferation and angiogenesis of endothelial cells in CRC (Cho et al., 2019). Isoquercitrin (IQ) is the main flavonol in *PM* fruits, which effectively inhibits the proliferation of SK-MEL-2 skin cancer cells. At the same time, the same concentration of IQ has no cytotoxic effect on human keratinocytes HaCaT (Won et al., 2020). However, very few studies have investigated the treatment of CRC with *PM* and its active ingredients roundly. Therefore, this article aims to explore the mechanism of *PM* in the treatment of CRC.

*RelA*/P65 is a member of the nuclear factor κB (NF-κB) transcription factor family, which is a class of proteins with specific DNA-binding activity that regulates a variety of functional target genes (Hoesel and Schmid 2013). NF-κB is a class of nuclear proteins that combine with the response element of immunoglobulin K enhancer to regulate the biological function of κB light chain in B cells (Sen and Baltimore 2006). External stimuli gather together to activate the upstream IκB kinase (IKK) complex, resulting in the release of NF-κBs into the nucleus to play a role in transcriptional regulation. Dimerization of NF-κB family members mediated by Rel homologous domain (RHD) is a necessary condition for DNA binding, and the up-regulation of gene expression depends on the transcriptional activation domain of *RelA*, C-REL, and RelB (Lu and Yarbrough 2015). Thus, the posttranslational modification of *RelA* determines the functional activity of NF-κB. Increased NF-κB activity can be attributed to activation pathways treated by mutated NF-κB genes or oncogenes and increased stimulators in the tumor immune microenvironment (Ben-Neriah and Karin 2011). Various adverse factors promote the high expression of NF-κB, which is a negative signal to the body, leading to tissue damage, immune disorders, and cancer (Staudt 2010). Activated NF-κB promotes CRC by accelerating cell proliferation and angiogenesis, inhibiting apoptosis, and promoting cell invasion and metastasis. The function of NF-κB in CRC is further complicated by the interaction of other signaling pathways. In short, NF-κB is involved in all stages of CRC development, from early adenoma to advanced cancer invasion and metastasis (Patel et al., 2018). It is also demonstrated that *RelA* is engaged in the whole process of CRC.

**FIGURE 1:**
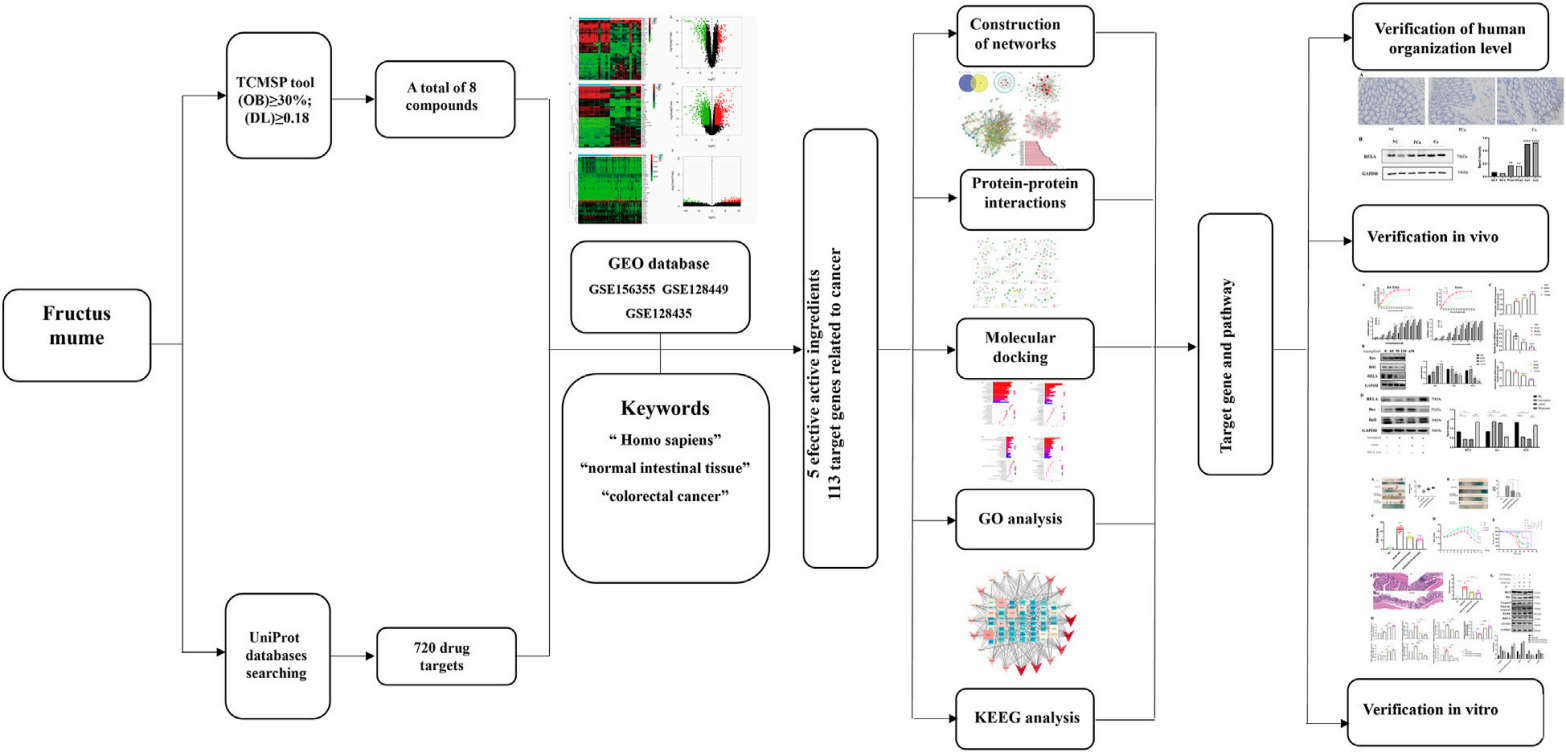
Network pharmacological, molecular docking, and experience verification workflow for identifying *PM* targets in the treatment of colorectal cancer.

In this study, the mechanism of and its bioactive component in the treatment of CRC was investigated by means of network pharmacology, molecular docking, and experimental validation. Figure 1 is the flowchart of this study. The function of *RelA* and the role of the NF-κB pathway in the treatment of CRC were investigated. *PM* and its extract are expected to be an effective drug in the treatment of CRC.

## Materials and Methods

### Chemical Composition Database and Active Compound Screening

We searched the critical word “*Prunus mume*” in the Traditional Chinese Medicine Database and Analysis Platform (TCMSP) website and the TCM Integrative Database (www.megabionet.org/tcmid/). The bioavailability (OB) >30% and drug similarity (DL) >0.18, main pharmacokinetic parameters, were used to screen the active components of *PM* using the pharmacokinetic indexes of absorption, distribution, metabolism, and excretion (ADME) (Liu et al., 2013; Shen et al., 2017; He R. et al., 2020). The OB value mainly reflects the proportion that a drug can overcome a series of barriers to be absorbed by the body and enter the blood circulation. The DL value mainly reflects the degree of similarity between the target molecule and the proven drug in composition, which can remove the components that are not suitable for the drug in chemical composition (Shen et al., 2017; He T. et al., 2020). The combination of the two values is used to screen drug components. Then, the target genes corresponding to the active ingredients of the drug were retrieved using the TCMSP (https://tcmsp-e.com/).

### Acquisition of Critical Genes Associated With CRC

The index words, “*Homo sapiens*,” “normal intestinal tissue,” and “colorectal cancer,” were used as keywords in the GEO database. Genes expressed differently between CRC tissues and normal tissues were sorted out and classified into functional groups with high expression and low expression (GSE156355, GSE128449, and GSE128435). The obtained gene list was imported into the Uniprot database (http://www.uniprot.org/) to bring the name and gene ID (He R. et al., 2020).

### Construction of Networks

Drugs’ active ingredients, the corresponding target genes of active ingredients, and disease screening target genes form the building “drug–disease” common target genes together, which build plum-visual networks of CRC target genes by Cytoscape 3.7.0 (http://www.cytoscape.org/). In the network diagram, nodes represent the active components or gene/proteins of *PM*, and interactions between nodes are represented by lines.

### Analysis of Protein–Protein Interaction Network

The “drug–disease” target genes were imported into the STRING biological database (https://string-db.org/) to study the target protein interaction relationship, also known as protein–protein interaction (PPI). All targets were limited to the species *H. sapiens*. In the PPI diagram, each solid circle depicts a gene, and the middle of the circle represents the protein structure. The circles are connected by different-color lines, and the strength of the interaction relationship is indicated by the number of connecting lines. At the same time, the bioinformatics technology was used to verify the protein interaction relationship again and draw the PPI interaction graph.

### Enrichment Analysis for Target Proteins

By bioinformatics technology, with its programming packets and Bioconductor high-throughput genomic data analysis (http://www.bioconductor.org/), Gene Ontology (GO) enrichment analysis and Kyoto Encyclopedia of Genes and Genomes (KEGG) pathway analysis were performed to extract the biological functions of target proteins and cancer-related pathways. *PM* plays an anti-CRC role by regulating the biological functions of target proteins or related signaling pathways. At the same time, the histogram and bubble graph are drawn to visualize the data.

### Molecular Docking

Using molecular docking technology to study the active components of *PM* and their related targets for CRC treatment can explain the mechanism of action and binding activity between the active components and target proteins to some extent. Compound structures in SDF format downloaded from the PubChem database (https://pubchem.ncbi.nlm.nih.gov/) were converted to a mol2 format file using Chem 3D software. The PDB format structures of BAX (PDB ID: 4S0O), BCL2 (PDB ID: 4LXD), CASP3 (PDB ID: 4PS0), CCND1 (PDB ID: 2W96), *EGFR* (PDB ID: 4I24), MAPK8 (PDB ID: 2XRW), *RELA* (PDB ID: 3QXY), and VEGFA (PDB ID: 1MKK) were downloaded from the RCSB Database (HTTPS://www.rcsb.org/), using Pymol software to remove solvent molecules and ligands, using AutoDock Tools 1.5.6 software to hydrogenate, calculate charge, assign atomic types, and so on and saving them as PDBQT format. Finally, Autodock Vina 1.1.2 was run for molecular docking, and Discovery Studio 2020 was used to analyze the conformation of docking visually. According to the data of intermolecular binding energy, hydrophilic and hydrophobic interaction, and electrostatic interaction, the score was given comprehensively, and then the intermolecular interaction was verified by next experiments.

### Experimental Validation of *PM* in Colorectal Cancer

The experiments involving human tissues have been carried out in accordance with The Code of Ethics of the World Medical Association (Declaration of Helsinki) for experiments involving humans and uniform requirements for manuscripts submitted to biomedical journals. All animal experiments should be carried out in accordance with the National Institutes of Health guide for the Care and Use of Laboratory animals (NIH publication no. 8023, revised 1978). All animal studies complied with the ARRIVE guidelines. All of the following tissue and animal experiments have passed the ethical review of Huazhong University of Science and Technology and Wuhan Union Hospital.

#### Preparation of *PM* Extract

Plant extract takes plants as raw materials, and according to the needs of the final product extracted, it is obtained and concentrated through the physical and chemical extraction and separation process. A product formed by one or more of the active ingredients in a substance without changing the structure of the active ingredients. Wumei is extracted on a proportion, which is the powder made after the extraction and concentration of plants raw materials. The means of proportion is the mathematical proportion of the amount of raw materials before extraction and the product after extraction and concentration. Proportional extracts generally do not have a very specific composition and content. Wumei was extracted from 10 kg of raw material and condensed into 1 kg of powder product. *PM* was purchased from Chengdu Effa Biological Technology Co., Ltd. (Chengdu, China). Certificate of analysis is shown in Supplementary Figure 2. The species was identified by Prof. Bo Liu from Changchun University of Chinese Medicine (Changchun, China). *PM* was dried and extracted in 75% ethanol in a shake incubator (Shanghai Jiecheng Experimental Instrument Co., Ltd., Shanghai, China) overnight at room temperature and filtered with Whatman Quantitative Filter Paper. The extracts of *PM* (E*PM*) were concentrated using a rotary vacuum evaporator (GongyiYuhua instrument Co., Ltd., Gongyi, China).

#### Cell Culture and Transfection

The CRC cell lines of HCT116 and Lovo were purchased from the American Type Culture Collection (ATCC, Manassas, VA, United States). The culture media was Dulbecco modified eagle medium, supplemented with 10% fetal bovine serum. The cells were put in an incubator (37°C, 5% CO_2_). Cell lines were authenticated by short tandem repeat analysis using the GenePrint 10 System at the Australian Genome Research Facility and confirmed to be *Mycoplasma*-free using the Lookout Mycoplasma PCR Detection Kit.

#### Overexpression Plasmid Transfection

The overexpression plasmid transfection of *RelA* and the negative control plasmid were designed and synthesized by Nanjing Norvizan Biological Technology Co., Ltd. (Nanjing, China). When the cells reached 70%–80% confluence, we performed siRNA transfection using Lipofectamine® RNAiMAX Transfection Reagen (Invitrogen, United States) strictly under the manufacturer’s instructions.

#### Establishment of the Azoxymethane/Dextran Sulfate Sodium Animal Model and *PM* Treatment

Sixty C57BL/6 wild-type age-matched (8–9 weeks) male mice (The Beijing Vitong Lihua Experimental Animal Technology Co. Ltd.) were used for this study. Forty-five mice were injected intraperitoneally (i.p.) with 12 mg/kg azoxymethane (AOM) and 3% dextran sulfate sodium (DSS). Starting at week 2, the mice were given 3% DSS three times a week (Tuesdays, Thursdays, and Saturdays) by intragastric administration for 1 week, followed by regular water for 2 weeks and repeated three cycles for 9 weeks. The rest of the 15 mice were injected i.p. with only 0.9% normal saline in week 1 served as untreated healthy controls. The AOM-injected mice were further subdivided into three groups of 15 mice. Group 1 (model group): Except for the above operation, the mice were given a regular diet; group 2 (*PM* 200 mg/kg group): except for the above operation, the mice were given 200 mg/kg *PM* three times a week (Monday, Wednesday, Friday) by intragastric administration for 9 weeks; group 3 (*PM* 400 mg/kg group): except for the above operation, the mice were given 400 mg/kg *PM* three times a week (Monday, Wednesday, Friday) by intragastric administration for 9 weeks. All mice were killed by cervical dislocation in week 10, and the number of macroscopic colonic tumors and the length of colorectum of the four groups were analyzed. Animal experiments were conducted following internationally recognized experimental animal management standards and approved by the Animal Ethics Committee of Huazhong University of Science and Technology (Wuhan, China) with *ad libitum* access to water and standard rodent diet.

The mice were maintained under controlled temperature and a 12-h light–dark cycle. All animal procedures were strictly carried out under the approved guidelines.

#### Cell Counting Kit-8 Assay

Cell viability was analyzed by Cell Counting Kit-8 (CCK8; Beyotime, Shanghai, China), according to the manufacturer’s protocols. Cells were seeded and cultured at a density of 5 × 10^3^/well in 100 μL of the medium into 96-well microplates (Corning, United States). Then, the cells were treated with various concentrations of kaempferol (0, 15, 30, 60, 90, 120, 150, 240 μM). After treatment for 6, 12, 24, and 48 h; 10 μL of CCK-8 reagent was added to each well and then cultured for 2 h. All experiments were performed in triplicate. The absorbance was analyzed at 450 nm using a microplate reader (Bio-Rad, Hercules, CA, United States) using wells without cells as blanks. The proliferation of cells was expressed by absorbance.

#### Western Blot Analysis

Total protein was extracted from the tissues and cell lines using tissue extraction reagent and cell extraction buffer (Beyotime) purified and assessed qualitatively by Western blot (WB) analysis. Total protein extracts (30 µg) were shifted to 12% gradient sodium dodecyl sulfate–polyacrylamide gel electrophoresis gels to separate the proteins by different molecular weights and transferred onto nitrocellulose membranes for antigen–antibody reaction. The membranes were then blocked with 5% skimmed milk in TBST for 1 h and mixed with primary antibodies against *RELA* (1:1,000; ABclonal), Bax (1:1,500; ABclonal), Bcl2 (1:1,000; ABclonal), caspase 3 (1:1,000; ABclonal), cleaved caspase 3 (1:1,000; ABclonal), *EGFR* (1:1,000; ABclonal), CCND1(1:1,000; ABclonal), and GAPDH (1:1,000; ABclonal) and then incubated with goat anti-rabbit secondary antibodies (ABclonal) for 2 h at 37°C (Jiang et al., 2018).

#### RNA Extraction and Quantitative Real-Time Polymerase Chain Reaction

Total RNA was extracted from the cancer tissues, matched normal tissues and cell lines. Total RNA was prepared using Trizol (Takara), and the cDNAs were generated by PrimeScript™ RT reagent kit according to the manufacturer’s instructions. The relative mRNA expression of GAPDH (mouse), *RELA* (mouse), Bax (mouse), Bcl2 (mouse), caspase 3 (mouse), cleaved caspase 3 (mouse), *EGFR* (mouse), CCND1(mouse), GAPDH (human), *RELA* (human), Bax (human) and Bcl2 (human) were executed using specific primers and the QuantiTest SYBR-Green polymerase chain reaction (PCR) kit (Norvizan Biological Technology Co., Ltd., Nanjing, China) on an Applied Biosystems 7500 real-time PCR machine (Applied Biosystems, Foster City, CA, United States). The GAPDH acted as a normalization control for all of the mRNAs listed above. The primers for qRT-PCR are shown in Table 1.

**TABLE 1:** qPCR primer sequence list of related genes.

Gene	Sequences of primers	
GAPDH	Forward	5′-AAC​CTT​CAG​ATG​CTG​CCA-3′
	Reverse	5′-AAA​CAC​ACA​GTC​ATC​ATA​GGG-3′
RelA	Forward	5′-TGC​AAG​ACT​CAT​CGA​CAA​GG-3′
	Reverse	5′-AGG​GGA​TTC​AAC​ATC​AGT​GC-3′
Bax	Forward	5′-TCT​ACT​TTG​CCA​GCA​AAC​TGG​TGC-3′
	Reverse	5′-TGT​CCA​GCC​CAT​GAT​GGT​TCT​GAT-3′
Bcl2	Forward	5′-ATG​ACC​AGA​CAC​TGA​CCA​TCC​ACT-3′
	Reverse	5′-ATG​TAG​TGG​TTC​TCC​TGG​TGG​CAA-3′
Caspase 3	Forward	5′-GAA​AGC​CGA​AAC​TCT​TCA​TCA​T-3′
	Reverse	5′-ATG​CCA​TAT​CAT​CGT​CAG​TTC​C-3′
Cleaved caspase 3	Forward	5′-TGC​ATA​CTC​CAC​AGC​ACC​TGG​TTA-3′
	Reverse	5′-CAT​GGC​ACA​AAG​CGA​CTG​GAT​GAA-3′
EGFR	Forward	5′-TTC​AGT​CAC​ATG​CTG​CTT​CC-3′
	Reverse	5′-GGC​TGC​TGT​CCT​ACC​AGA​CT-3′
CCND1	Forward	5′-AAA​ACA​UAG​AAA​AAU​UCA​GCA​A-3′
	Reverse	5′-CAU​GUG​GUC​UGU​CGC​AUA​AUA-3′

#### Immunohistochemistry

Formalin-fixed tissues were embedded in paraffin and sliced. The 4-mm-thick sections were then incubated with monoclonal antibodies against *RELA* (1:100; ABclonal) overnight at 4°C and then incubated with goat anti-rabbit secondary antibodies (Abcam, United States). 3,3′-Diaminobenzidine was used as chromogen. The sections were then dyed using hematoxylin and mounted. *RELA* expression was evaluated qualitatively by two independent laboratory technicians.

### Statistical Analysis

Based on three independent experiments, data are expressed as mean ± SEM, and statistical analyses were performed using SPSS 18.0 software, R software version 3.5.0 (R Foundation for Statistical Computing, Vienna, Austria), and GraphPad Prism version 8.0.1 (GraphPad Software Inc., United States). Significant differences were considered if the probability of the difference was <5 in 100 (*p* < 0.05).

## Results

### Screening Bioactive Components of *PM* and Differential Gene Expressions in CRC

**FIGURE 2:**
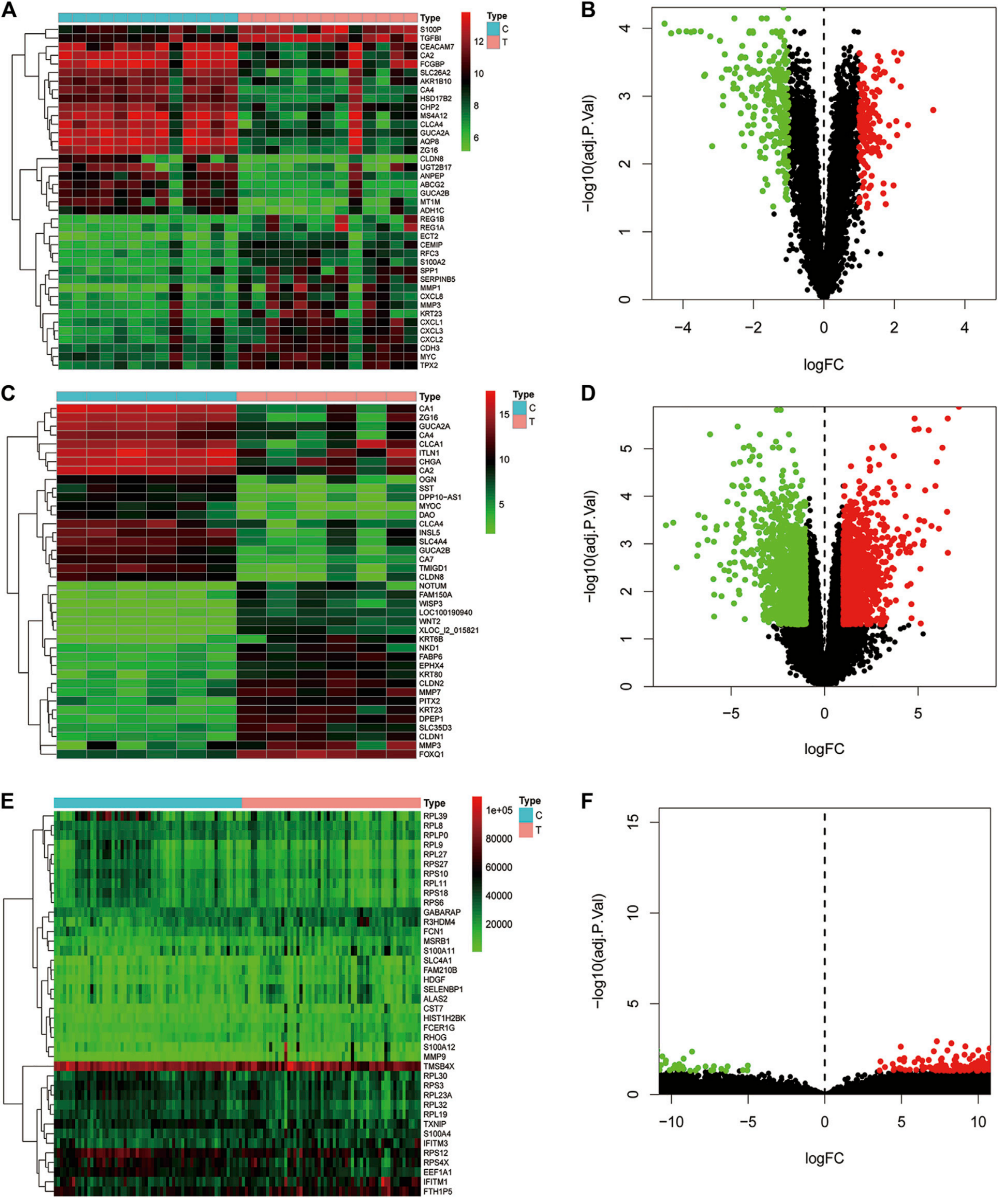
Analysis of differential gene expression in three datasets associated with colorectal cancer and normal tissue (GSE164191, GSE110225, and GSE156355) in GEO database. A total of 6,649, 406, 2954 differentially expressed genes were screened from normal tissues and colorectal cancer tissues, respectively. Heat maps and scatter maps were drawn according to different expression levels of differentially expressed genes, and some differentially expressed genes were selected for display. In the figure, red represents high expression, and green represents low expression. **(A**, **B)** Heat map and scatter map of differential genes in GSE164191 dataset. **(C**, **D)** Heat map and scatter diagram of differential genes in GSE110225 dataset. **(E**, **F)** Heat map and scatter diagram of differential genes in GSE156355 dataset.

Data of *PM* and differential gene expression (DGE) in CRC were obtained from TCMSP (https://old.tcmsp-e.com/tcmsp.php) and GEO (https://www.ncbi.nlm.nih.gov/geo/) databases. The 40 known components and 720 drug target indexes of *PM* were obtained by TCMSP database retrieval. A total of eight compounds were obtained from the TCMSP database under the screening conditions of OB ≥30% and DL ≥0.18 (Table 2). The corresponding genes were converted into GeneSymbol through the Uniprot database, and then duplicated and nonhuman targets were deleted; 170 drug targets were finally obtained. The two-dimensional and three-dimensional structures of protein molecules were retrieved by the PubChem database (https://pubchem.ncbi.nlm.nih.gov/) (Table 2). Differential genes were screened out by processing of three datasets, GSE164191, GSE110225, and GSE156355 in the GEO database. DGE analysis was conducted on the three datasets respectively, and the results are shown in Figure 2. A total of 10,009 genes were screened, among which 8,981 target genes were screened by removing duplicate, meaningless, and blank data.

**TABLE 2:** Eight main active ingredients of *PM* screened from TCMSP database.

Mol ID	Molecule name	Two-dimensional structure	Three-dimensional structure	BO (%)	DL
MOL001040	(2R)-5,7-dihydroxy-2-(4-hydroxyphenyl)chroman-4-one	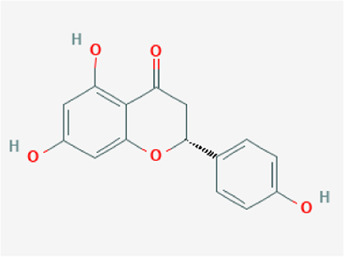	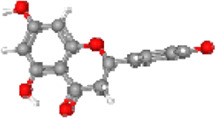	42.36	0.21
MOL000358	β-Sitosterol	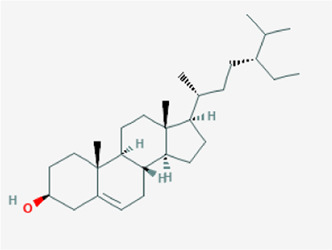	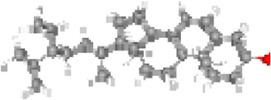	36.91	0.75
MOL000422	Kaempferol	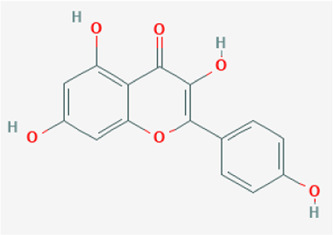	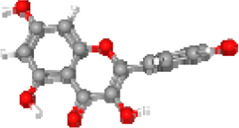	41.88	0.24
MOL000449	Stigmasterol	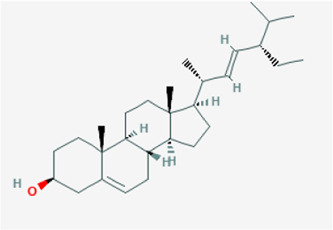	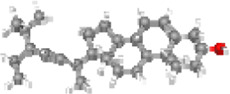	43.83	0.76
MOL005043	Campest-5-en-3beta-ol	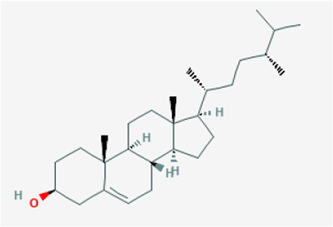	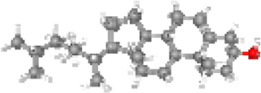	37.58	0.71
MOL008601	Methyl arachidonate	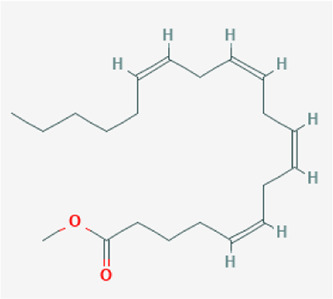	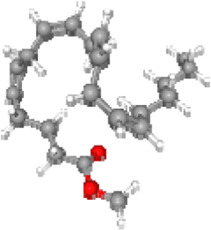	46.90	0.23
MOL000953	CLR	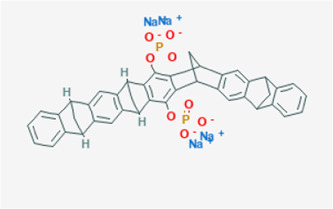	-	37.87	0.68
MOL000098	Quercetin	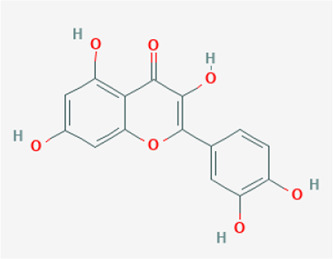	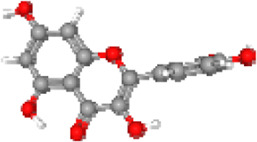	46.43	0.28

### Construction and Analysis of *PM* Targets–CRC Differential Gene Expression Network

**FIGURE 3:**
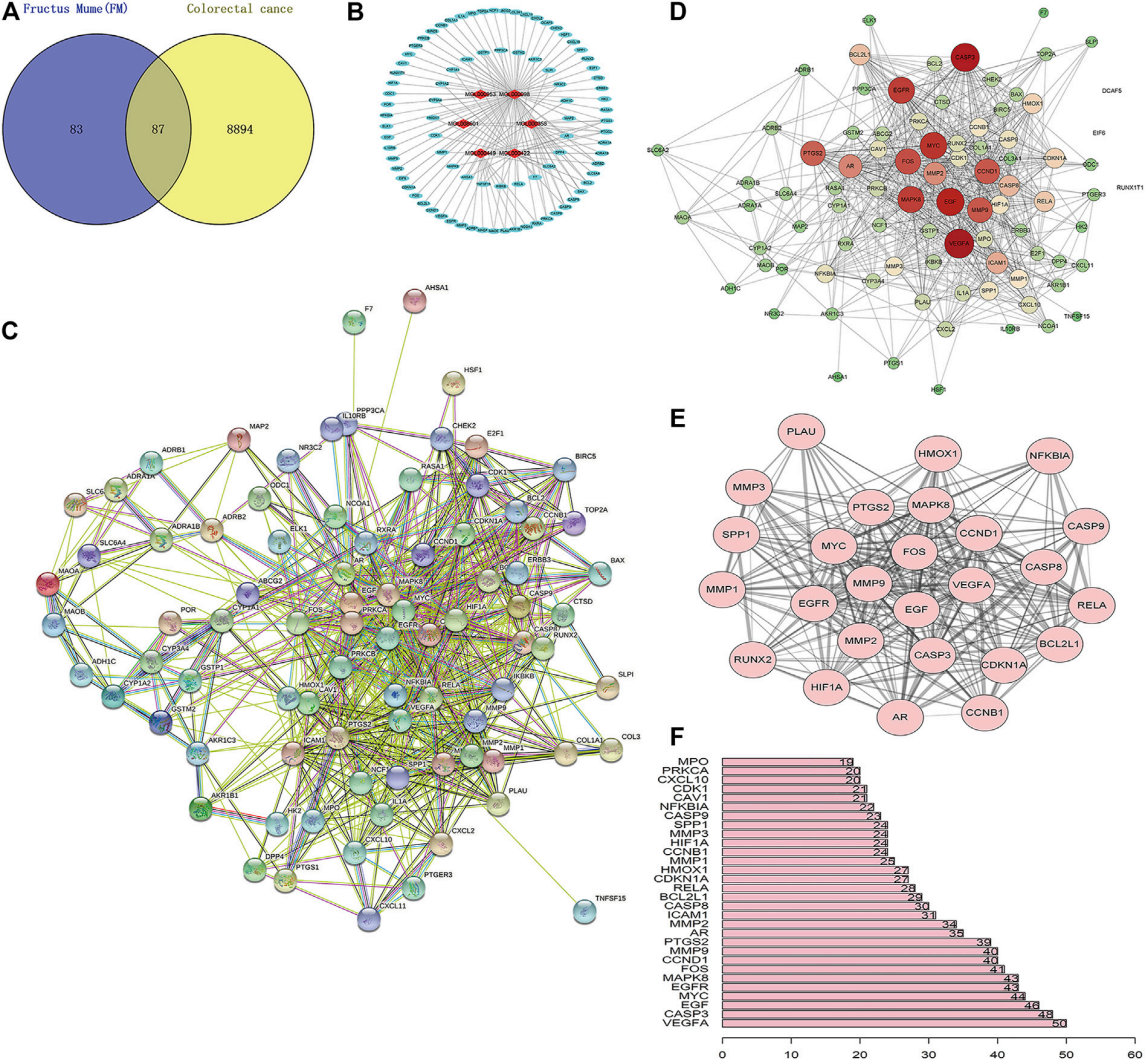
Construction of *PM* active ingredient—target gene network in colorectal cancer and screening of key molecules. **(A)** Venn diagrams of drug and disease targets. After screening and repeating, blue represents only the drug target, yellow represents only the disease target, and the middle color represents the intersection of the two targets. **(B)** Construction of drug active components—target gene network. **(C)** Target protein interaction network analysis (PPI) in STRING database. **(D)** Target protein interaction network analysis (PPI) in Cytoscape 3.8.2 software. **(E)** The top 25 genes with higher expression differences were obtained by PPI screening. **(F)** Protein interaction relationship histogram of *PM*.

A total of 170 *PM* targets and 8,981 CRC DGEs were comapped, common targets were obtained through the Venny 2.1 website, and Venn diagrams were drawn (Figure 3A). Through the docking and integration of drug targets and disease-related targets, a total of 87 drug–disease key targets were screened out. Eight active ingredients and 87 drug–disease targets were imported into Cytoscape 3.8.2 software to visualize the gene network relationship between *PM* active ingredients and target genes (Figure 3B). As shown in Figure 3B, there are 93 nodes and 130 relation lines; The red rhomboid nodules are the active components of *PM*. The light blue oval node is the target gene of drug disease. Each active ingredient acts on at least one target gene, and each gene is regulated by at least two active ingredients, indicating the multitarget action characteristics of multiactive components of *PM*. The active ingredients with the most target genes in *PM* are Mol000098 (quercetin), Mol000422 (kaempferol), Mol000358 (β-sitosterol), and MOL000449 (stigmasterol). This study speculated that these active ingredients might be the fundamental constituents for *PM* treatment of CRC.

### Target Protein Interaction Network Analysis (PPI)

The STRING database was utilized to construct *PM* target–CRC DGE network. *PM*-CRC target genes were imported, the species selected was *H. sapiens* with a combined score of ≥0.4, and the network diagram of protein interaction was obtained (Figure 3C). As shown in Figure 3C, there are 166 different-color circles, each representing all the proteins encoded by a target gene, 2,361 interacting relationship lines. Each line represents a protein–protein association, representing that two proteins interact functionally, but not necessarily bind structurally. Meanwhile, Cytoscape 3.8.2 software was used to analyze further and score the *PM* target–CRC DGE network, and the molecular network diagram as shown in Figure 3D was obtained. Statistical analysis was carried out on each target gene, and the top 30 proteins with the most significant number of adjacent genes were selected for statistical analysis (Figure 3E). The proteins whose connection degree is for more than 40 contain VEGFA, CASP3, EGF, *EGFR*, MYC, MAPK8, FOS, CCND1, and MMP9 (Figure 3F). Other molecules with high connectivity obtained HIF1A, *RelA*, and Bcl2 through PPI screening (Figure 3E). Genes with more linked proteins play a more critical role in the network, play a more pronounced role in *PM*-CRC correlation, and are more likely to become therapeutic targets of drugs acting on diseases.

### Predicting Functional Enrichment Analysis for *PM*

**FIGURE 4:**
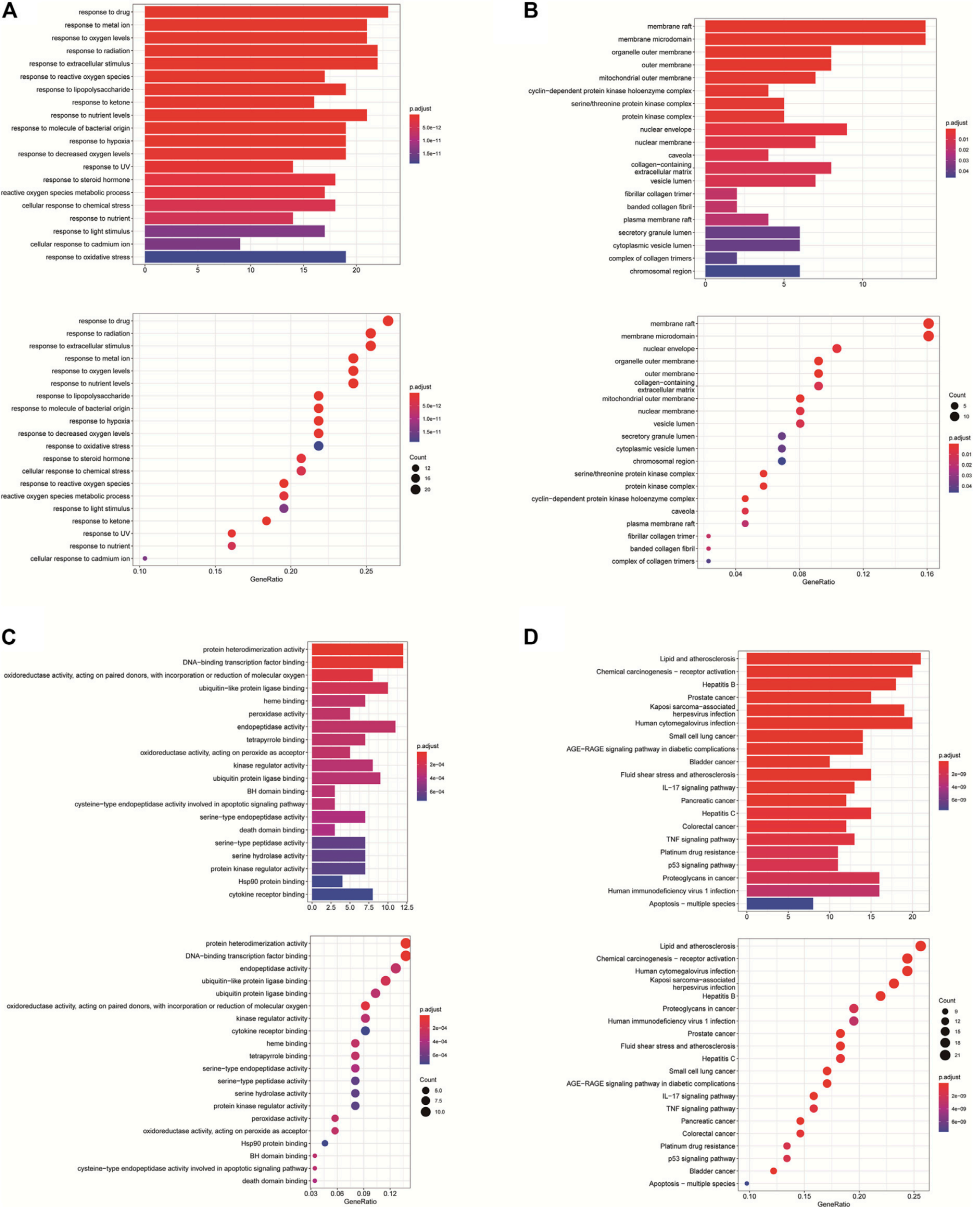
Enrichment analysis of Gene Ontology (GO) and Kyoto Encyclopedia of Genes and Genomes (KEGG) pathway biological process of anticolorectal cancer targets gene from active ingredients of *PM*. The color scale indicates the *p* value, and the dot size indicates the gene count in each term. **(A)** GO analysis of multicellular organismal development (BP), mitotic G1 DNA damage checkpoint, G1 DNA damage checkpoint, and mitotic G1/S transition checkpoint were screened out. **(B)** GO analysis of intracellular membrane-bounded organelles (CC), cyclin-dependent protein kinase holoenzyme complex, serine/threonine kinase complex and protein kinase complex were screened out; **(C)** GO analysis of protein binding (MF). **(D)** KEGG analysis, CXCR chemokine receptor binding, and sulfur compound binding were screened out.

**FIGURE 5:**
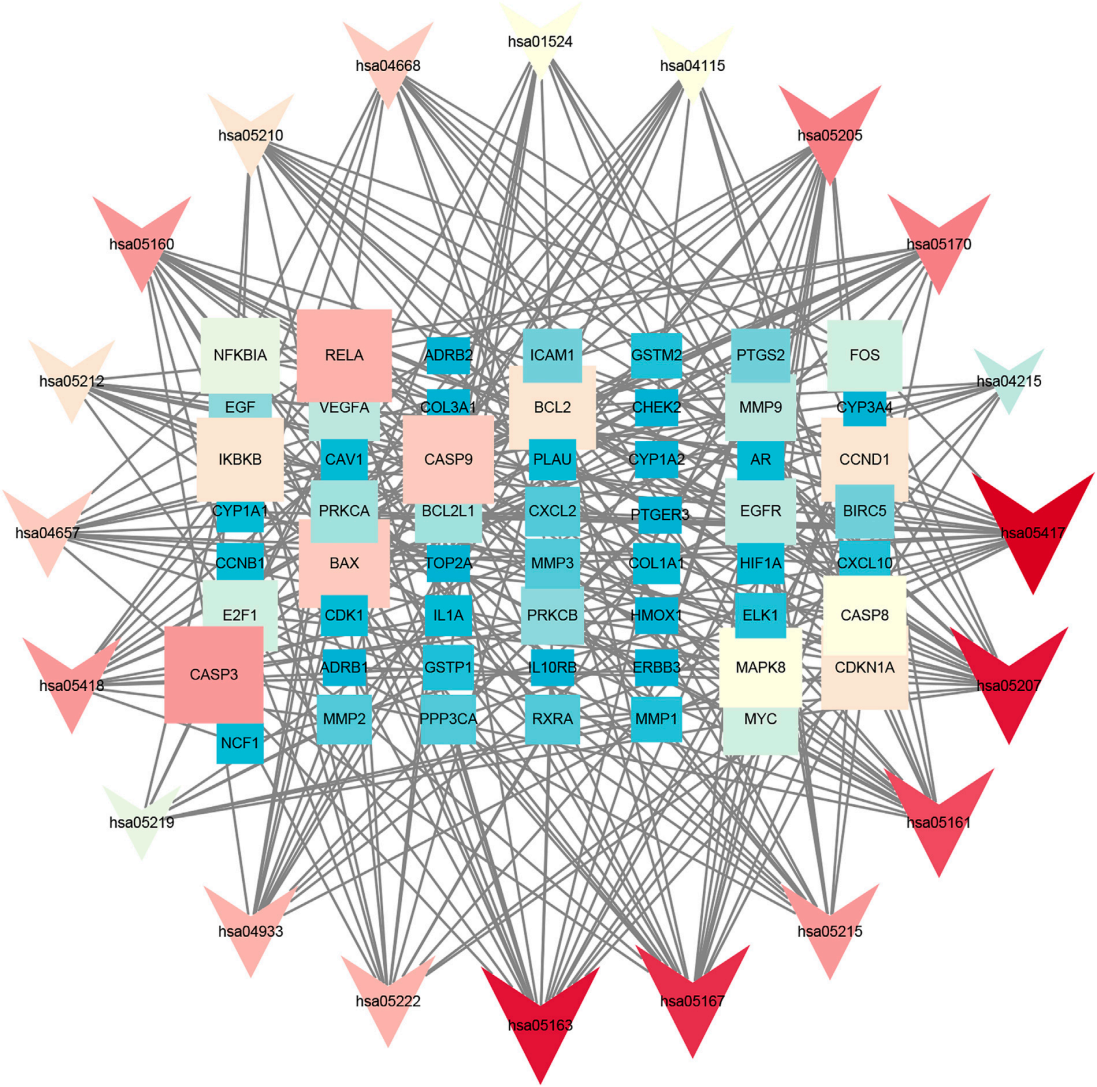
Kyoto Encyclopedia of Genes and Genomes (KEGG) analysis in Cytoscape 3.8.2 software. The KEGG screening was conducted using Cytoscape 3.8.2 software. The association network between target genes and pathways was constructed. The target genes and KEGG pathways were scored according to the connectivity degree. A total of eight high expression of target protein include CASP3, BAX, RELA, EGFR, BCL2, CCND1, MAPK8, and VEGFA. The pathway involved in this process mainly includes the apoptosis pathway by KEGG analysis.

GO annotation analysis showed that drug–disease target proteins were mainly involved in protein heterodimerization activity, DNA-binding transcription factor binding, and oxidoreductase activity, acting on paired donors, with incorporation or reduction of molecular oxygen and ubiquitin-like protein ligase–binding functional activities (Figures 4A–C). Meanwhile, KEGG enrichment analysis showed many target genes were closely related to lipid and atherosclerosis, chemical carcinogenesis-receptor activation, human cytomegalovirus infection, Kaposi sarcoma–associated herpesvirus infection, and otitis B (Figure 4D). Drug therapy often works at the cellular and molecular levels through a network of multiple pathways and multiple targets. Among these potential targets, *RELA* (*RELA* proto-oncogene, NF-κB subunit), CASP3, CASP9, BAX, BCL2, CCND1, and CDKNIA were screened as relatively high-level targets (Figure 5), which played an essential role in the inhibition effect of *PM* on CRC. We hypothesized that *PM* plays an inhibitory role in CRC by regulating *RELA* and above other molecules. That NF-κB pathway is involved in the treatment of CRC by *PM*, which may play an antitumor role by promoting the apoptosis of tumor cells.

### Molecular Docking Verified the Interaction Between *PM* Active Components and Target Proteins

**FIGURE 6:**
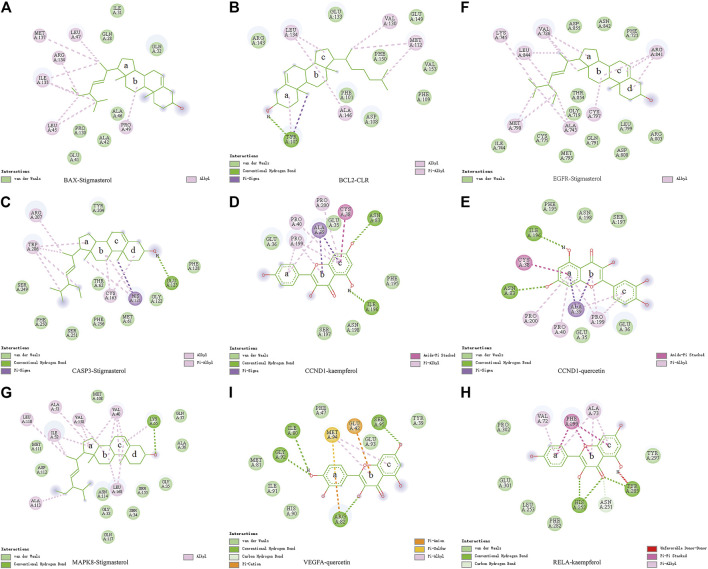
Binding of *PM* compounds in interaction with target proteins. The bingding mode of *PM* compounds, including stigmasterol, CLR, kaempferol, and quercetin in the proteins **(A)** BAX, **(B)** BCL2, **(C)** CASP3, **(D,E)** CCND1, **(F)** EGFR, **(G)** MAPK8, **(H)** RELA, and **(I)** VEGFA.

Molecular docking was used to verify if the top five compounds had a significant role in regulating above eight DGEs. The interaction was scored, and the results are shown in Table 3. According to the scoring results, stigmasterol-BAX, stigmasterol-CASP3, stigmasterol-*EGFR*, stigmasterol-MAPK8, kaempferol-*RELA*, kaempferol-CCND1, quercetin-CCND1, quercetin-VEGFA, and CLR-BCL2 had a strong correlation, among which kaempferol-*RELA* had the highest correlation score. Further experiments are needed to verify the strong relationship in CRC. The molecular docking results are shown in Figure 6, which shows that all the essential compounds in the network had a strong affinity with corresponding DGEs.

**TABLE 3:** Docking scores of eight major highly expressed molecules with major active components.

Protein	Active ingredients	Affinity (kcal/mol)
BAX	Stigmasterol	−8.5
BCL2	CLR	−8
CASP3	Stigmasterol	−7.5
CCND1	Kaempferol	−6.1
	Quercetin	−6.1
EGFR	Stigmasterol	−9.1
MAPK8	Stigmasterol	−8.9
RELA	Kaempferol	−9.4
VEGFA	Quercetin	−6.4

### Experimental Validation

#### *RELA* Highly Expresses in Colorectal Tissue

**FIGURE 7:**
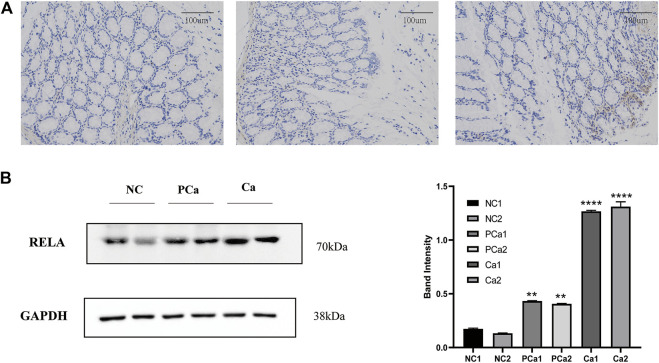
The RELA molecule was highly expressed in human colorectal cancer tissue samples. **(A)** Images depicting immunohistochemical (IHC) staining of RelA conducted in tissue sections derived from human normal colon, paracarcinoma tissues, and colon cancer samples. Images were captured at ×200 magnification. **(B)** Western blot and gray value statistics of RelA in human colon tissue samples. NC, normal colon of the patient; PCa, paracarcinoma tissues of the patient; Ca, patient colon cancer tissue. *p < 0.05 vs. negative control (NC) group, **p < 0.02 vs. NC group, ***p < 0.01 vs. NC group, ****p < 0.001 vs. NC group.

NF-κB is a crucial transcription factor in the inflammatory tumor microenvironment, and *RelA*/P65 is center to the activation of Wnt signaling by NF-κB, which is one of the center mechanisms of tumor cell initiation in CRC (Schwitalla et al., 2013). To verify the expression of *RelA* in colon cancer tissue, we conducted immunohistochemical analyses in average, paracancer, and colon cancer tissue samples from the patients. The presentation of *RelA* in colon cancer specimens was significantly higher than that in paracancer and standard specimens (Figure 7A). We randomly selected two groups from 16 groups of tissue samples provided by the Department of Gastrointestinal Surgery of Wuhan Union Hospital for a WB test. WB result and gray value statistics of *RELA* in human colon tissue samples showed that *RELA* is highly expressed in colonic adenocarcinoma tissues (Figure 7B). The above results indicate that *RELA* is highly expressed in human colon tissue, and *RELA* can be used as a marker of CRC formation.

#### *PM* Extract Suppressed CRC *In Vivo* by Promoting Apoptosis via Inhibiting the Expression Level of *RELA* Protein

**FIGURE 8:**
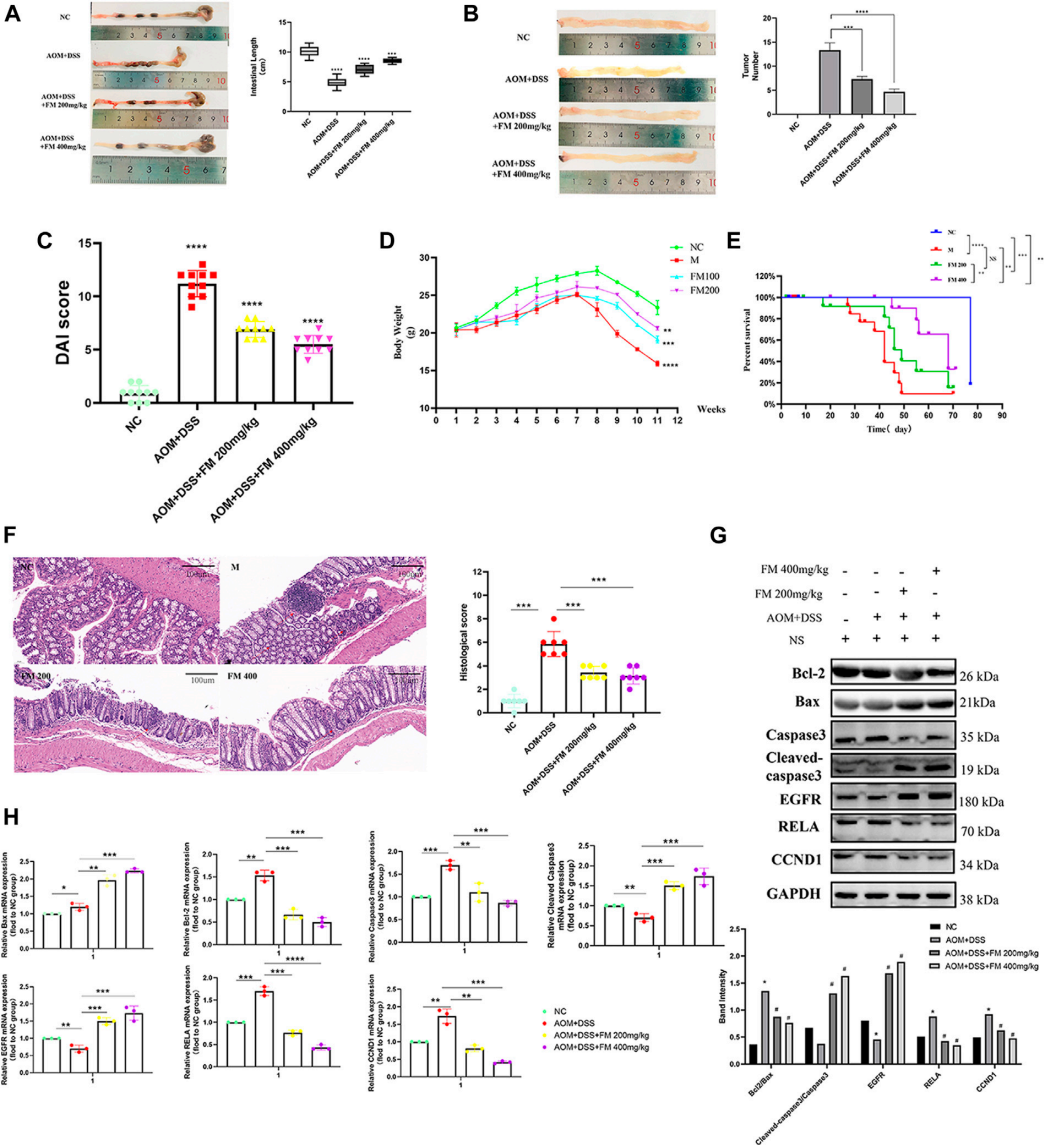
*PM* inhibits the progression of colorectal cancer in mice by promoting apoptosis. **(A)** *PM* improved intestinal shortening in colon cancer model mice. **(B)** *PM* inhibits intestinal tumorigenesis in colon cancer model mice. **(C)** *PM* improved intestinal DAI score in colon cancer model mice. **(D)** *PM* slowed weight loss in colon cancer model mice. **(E)** In survival analysis, *PM* improved survival in colon cancer model mice. **(F)** Hematoxylin-eosin staining experiment verified that *PM* improved the intestinal structure destroyed by AOM and DSS and inhibited the development of tumor tissues. The tumor inhibition effect of 400 mg/kg was higher than 200 mg/kg. **(G)** Western blot and **(H)** qPCR assays confirmed that *PM* inhibition of colon cancer in mice was mediated by apoptosis-related pathways. NC, normal group; M, model group, AOM + DSS; PM2, AOM + DSS + *PM* 200 mg/kg; PM4, AOM + DSS + *PM* 400 mg/kg. *p < 0.05 vs. negative control (NC) group, **p < 0.02 vs. NC group, ***p < 0.01 vs. NC group, ****p < 0.001 vs. NC group.

To further explore the effect of *PM* extract on CRC *in vivo*, we established the AOM/DSS–induced mouse CRC model and treated the mice with *PM* simultaneously. After 11 weeks, the mice in each group were sacrificed to obtain the entire colorectal tissue from the cecum to the end of the anus, conducting experimental validation at the organizational level. The results showed that the length of the CRC was significantly shortened in the AOM/DSS–induced model, and the shortening of the intestinal tract was improved considerably in the *PM*-treated group compared to that of the untreated model group. However, the improvement of colonic length was not apparent with the increasing concentration of *PM* extract (Figure 8A). The number of tumor formation per unit area of intestinal tissue is the indicator to measure the effect of modeling and treatment. *PM* extract significantly reduced the number of tumor formation per unit area (*p* < 0.05) and inhibited tumor formations in macroscopic perspective (Figure 8B). At the same time, the higher the concentration of *PM* extract, the more pronounced the inhibition of tumor formation, but the safety of a high concentration of *PM* extract for the body needs to be further verified. Compared with the control group, DAI (Disease Activity Index) score was significantly decreased (*p* < 0.01) (Figure 8C), weight was significantly increased (*p* < 0.01) (Figure 8D), and survival period was prolonged (*p* < 0.01) (Figure 8E) in the *PM* extract treatment group, which had not improved in the high-concentration group. The colon tissues were taken for hematoxylin-eosin staining. The results showed that the model group lost the normal tissue morphology, the intestinal villi were destroyed, and there were apparent nuclear enlargement, nuclear aggregation, and nuclear shrinkage. Treatment with *PM* extract restored part of the damaged intestinal structure and improved the above pathological changes (Figure 8F). Western blotting and quantitative PCR (qPCR) were performed to examine the expression levels of key regulators responsible for apoptosis and core molecules screened through network pharmacology. As shown in Figures 8G,H , *PM* extract inhibited the expression of *RelA*, Bcl2, caspase 3, and CCND1 and promoted apoptosis-related proteins Bax, cleaved caspase 3, and *EGFR*. These results confirmed that *PM* extract is mainly involved in inhibiting CRC through apoptosis-related pathways, especially the NF-κB pathway in which *RelA* is engaged. *RelA* is a vital molecule regulated by *PM*, and the NF-κB pathway involved by *RelA* is a momentous mechanism of *PM*’s treatment of CRC.

#### Kaempferol Inhibits the Growth of Colon Cancer Cells by Promoting *RELA*-Related Apoptotic Pathways

**FIGURE 9:**
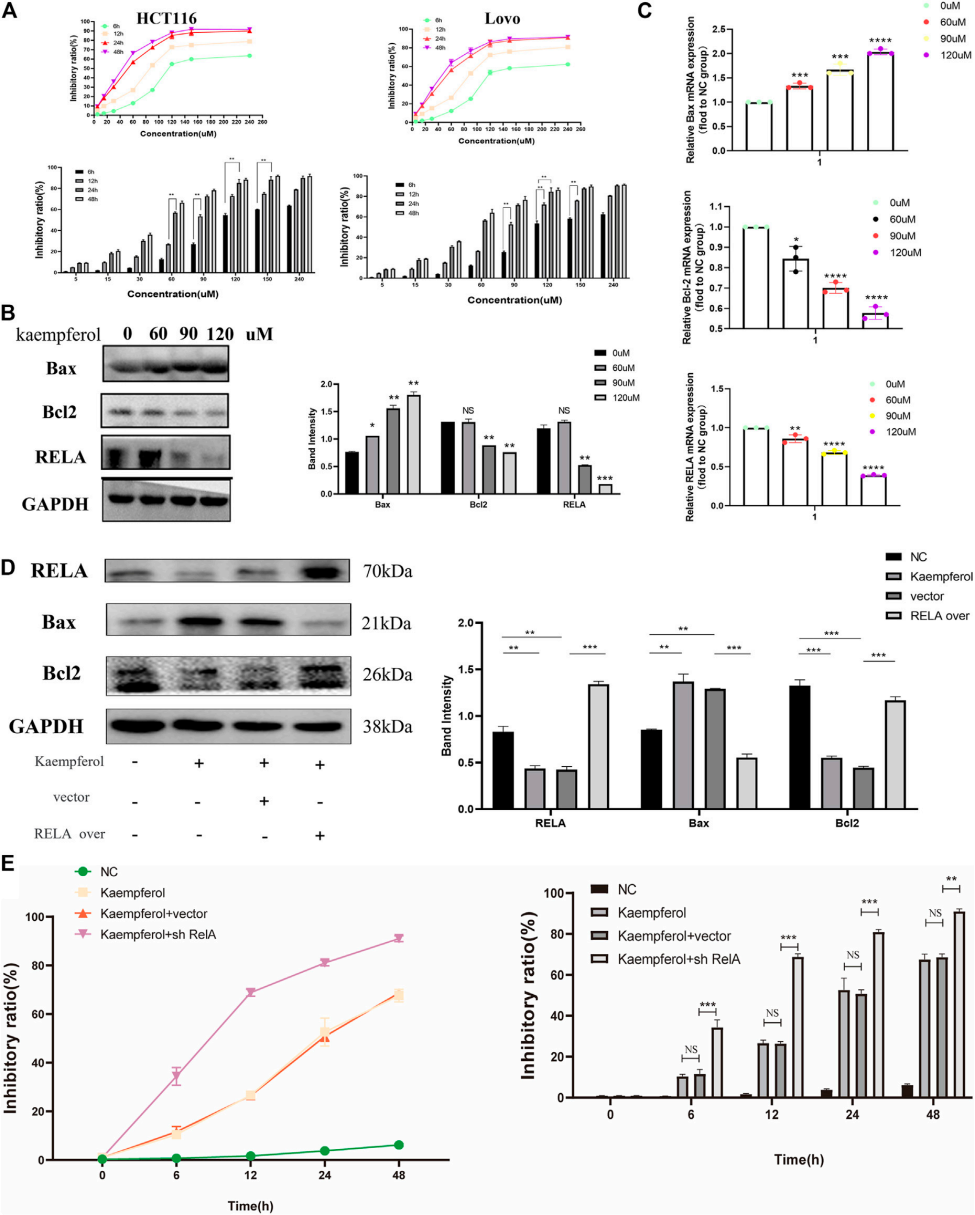
Kaempferol, an important active component in *PM*, inhibits the growth of colon cancer cells by promoting RELA-related apoptotic pathways. **(A)** CCK8 assay verified the effect of kaempferol on HCT116 and Lovo human colon cancer cell lines. Eight concentration gradients of 5, 15, 30, 60, 90, 120, 150, and 240 µM were implemented, and the results showed that the inhibition rates of the two cell lines were similar. The cells were significantly inhibited at the concentrations of 60, 90, and 120 µM. **(B)** Western blot assay and **(C)** qPCR assay verified that kaempferol regulated the expression of Bax, Bcl2, and RELA in HCT116 cells at concentrations of 60, 90, and 120 µM, respectively. RELA was significantly inhibited with the increase of the concentration. Apoptosis was increased. **(D)** After overexpressed RELA plasmid and no-loaded plasmid were transfected, the effects of kaempferol on the expression of RELA, Bax, and Bcl2 in HCT116 cell lines were confirmed by Western blotting. **(E)** After sh-RELA plasmid and no-loaded plasmid were transfected, the effects of kaempferol on the cell viability of HCT116 cell lines were confirmed by CCK8. *p < 0.05 vs. negative control (NC) group, **p < 0.02 vs. NC group, ***p < 0.01 vs. NC group, ****p < 0.001 vs. NC group.

Based on the above molecular docking results and previous literature reports, *RelA* was a potential target gene of kaempferol, one of the active components of *PM*, for the treatment of ulcerative colitis (Wei et al., 2020). Kaempferol could improve virus-induced acute lung injury through the NF-κB signaling pathway (Zhang et al., 2017). Therefore, we hypothesized that kaempferol might be involved in *PM* regulation of *RelA*, thus affecting the activity of CRC cells. Cell viability assays were performed on two human colon cancer cell lines HCT116 and Lovo, as shown in Figure 9A. These findings demonstrated that the activity of kaempferol on colon cancer cells was time- and dose-dependent. There was no difference between the two cell lines in IC_50_ value, which after 24 h of treatment was approximately 60 µM for the two cell lines. Moreover, after treating colon cancer cell HCT116 at concentrations of 60, 90, and 120 µM for 24 h by kaempferol, the expressions of *RelA* and apoptosis-related proteins Bax and Bcl2 were detected by WB. The results showed that kaempferol could inhibit the expression of *RelA* in colon cancer cell HCT116 (Figure 9B). At the same time, the effect of promoting apoptosis of tumor cells was more evident with the increase of the concentration. At the gene expression level, the expression of *RelA* and apoptosis-related proteins Bax and Bcl2 was detected by qPCR, and similar protein levels were obtained (Figure 9C). The above results only proved that kaempferol could change the expression of *RELA* in colon cancer cells, but did not indicate that kaempferol had a regulatory relationship with *RELA*. Therefore, we completed the cell transfection experiment to transfect the *RELA*-specific overexpression plasmid into HCT116 cells, and the results showed that in the case of *RelA* overexpression, the inhibitory effect of kaempferol on *RelA* was decreased, and the ability to induce apoptosis of tumor cells was weakened, which proved that kaempferol could regulate the expression of *RelA*, and *RelA* was the downstream target of kaempferol (Figure 9D). Kaempferol inhibits the expression of *RelA*, a crucial molecule in the NF-κB pathway, thereby promoting the apoptosis of colon cancer cells and inhibiting tumor growth.

## Discussion

In this study, we found that *PM* possesses an adequate therapeutic effect on CRC, primarily by inhibiting *RelA* expression and NF-κB pathway from promoting tumor apoptosis. On the GEO database, differentially expressed genes (DEGs) in CRC were identified. Simultaneously, we detected the bioactive components (β-sitosterol, kaempferol, stigmasterol, methyl arachidonate, and quercetin) and main target proteins related to CRC in *PM* using the TCMSP database. Subsequently, we further screened essential genes and core-dependent pathways through the PPI network, GO analysis, and KEGG analysis. Ultimately, the eight genes (*RELA*, CASP3, BAX, *EGFR*, BCL2, CCND1, MAPK8, and VEGFA) with the highest differential expression within the constraints and the apoptosis-associated pathway were identified. Then, we performed molecular fitting scoring and molecular docking for the eight genes and five bioactive components. The outcomes indicated that *RELA* and kaempferol were genes and bioactive compounds, respectively, with the best affinity and reasonable degree in *PM* for CRC treatment. The NF-κB transcription factors are known to consist of five homologous subunits (*RelA*/p65, C-rel, RelB, P50/NF-κB1, and P52/NF-κB2), which are usually dimerized and controlled by NF-κB inhibitors (IκBs) in the cytoplasm (Naugler and Karin 2008). Nuclear localization of *RelA*/P50 dimer is one of the regulatory factors of NF-κB canonical pathways, which is closely related to inflammatory tumorigenia (Annunziata et al., 2007; Keats et al., 2007). *RelA* had been used as an evaluator to evaluate the role of NF-κB activation in various tumors, including lung adenocarcinoma (He T. et al., 2020), breast cancer (Li et al., 2020), liver cancer (Moles et al., 2016), CRC (Liu et al., 2018b; Xu et al., 2019; Wang S. et al., 2020), and so on. In intestinal diseases, NF-κB is involved in the early formation of colorectal adenoma and may contribute to adenoma canceration (Shaked et al., 2012). At the mechanic level, *RelA*/NF-κB plays a crucial role in colorectal tumor–related processes such as angiogenesis, cell proliferation, apoptosis, autophagy, and metastasis (Ben-Neriah and Karin 2011). NF-κB represented antiapoptotic activity mediated by cFLIP, cIAP2, Bcl-XL, and Bcl2 and other antiapoptotic genes (Naugler and Karin 2008). Iκβ-mediated NF-κB activity had a cell-specific role in the development of colitis-associated cancers. NF-κB played a role in the early development of precancerous adenomas, which may be a potential target for clinical drug development and treatment (Greten et al., 2004; Hayakawa et al., 2009). Consequently, we hypothesized that *PM* inhibited the occurrence and growth of tumors by inhibiting the expression of *RELA*/NF-κB and the apoptosis-associated pathway.

The feasibility of *PM* in the treatment of human CRC from the perspective of morphology and protein expression was further proven (Figure 7), by verifying that the expression of *RelA* in human colon cancer tissues was indeed higher than that in normal tissues and paracancer tissues. Then we explored the potential mechanism of *PM* in the treatment of CRC, as it is well-known that inducing tumor cell apoptosis is a crucial therapeutic approach in the treatment of tumors. After applying kaempferol, apoptosis of HCT116 cells was significantly increased, and the expression of apoptosis-related proteins was increased.

The comparison results between the kaempferol treatment group and the *RelA* over with kaempferol treatment group showed that *PM* mainly affected apoptosis by inhibiting *RelA*. Our study showed that *RelA* overexpression could reduce the therapeutic effect of kaempferol, inhibit the expression of Bax, and promote the expression of Bcl2. Therefore, we can demonstrate that kaempferol, the bioactive component of *PM*, can promote the apoptosis of colon cancer cells by inhibiting *RelA*, thus playing a role in treating cancer. Simultaneously, we conducted experiments in mice to verify that *PM* improved the symptoms of CRC model mice and participated in regulating the expression of *RelA* and apoptosis-related proteins. *PM* extract inhibited the expression of *RelA*, Bcl2, caspase 3, and CCND1 and promoted apoptosis-related proteins Bax, cleaved caspase 3, and *EGFR*. These results were consistent with the predictions of network pharmacology. In addition, the binding force between a drug and its target protein is an important indicator to evaluate its mechanism of action on disease (Schmidt et al., 2010). We investigated the docking of five bioactive ingredients and eight proteins through molecular docking, and the results showed that kaempferol-*RelA* had the best docking effect. These results confirmed that *PM* extract is mainly involved in inhibiting CRC through apoptosis-related pathways, especially the NF-κB pathway in which *RelA* is engaged.

CHM was also verified as a regulator to improve the function of the immune system and inhibit tumor progression (Wang Z. et al., 2020). Many scholars believe that the bioactive components play a major role in anticancer. Among bioactive compounds of *PM*, β-sitosterol, kaempferol, stigmasterol, methyl arachidonate, and quercetin showed strong pharmacological effects. β-Sitosterol, kaempferol, and stigmasterol had obvious antioxidation, anti-inflammatory, antitumor, immunoregulation, and metabolic regulation activities (Wilt et al., 2000; Bin Sayeed and Ameen 2015; Devi et al., 2015; Eid and Haddad 2017; Imran et al., 2019; Reyes-Farias and Carrasco-Pozo 2019; Babu and Jayaraman 2020; Bae et al., 2020; Vo et al., 2020). β-Sitosterol interferes with multiple signaling pathways such as cell cycle, proliferation, apoptosis, inflammation, invasion, metastasis, and angiogenesis (Bin Sayeed and Ameen 2015). In terms of antitumor, β-sitosterol induced apoptosis by activating caspase-3 and caspase-9 and showed cytotoxic effects on HepG2 and Huh7 cells, but did not have an effect on human primary hepatic fibroblasts (Vo et al., 2020). Kaempferol reduced the risk of skin cancer, liver cancer, and colon cancer by blocking the cell cycle in the G2/M phase, down-regulating the related markers of epithelial–mesenchymal transition and accelerating cell necrosis and apoptosis (Imran et al., 2019). Stigmasterol had toxic effects on various tumors and restrained ES2 and OV90 cells by inhibiting cell migration and angiogenic genes in human ovarian cancer cells (Bae et al., 2020). Quercetin played an anticancer role by inducing autophagy, apoptosis, and inactivation of cells through MAPK/Erk1/2, PI3K/Akt/mTOR and Wnt/-catenin pathways (Reyes-Farias and Carrasco-Pozo 2019). Therefore, there is a theoretical basis for *PM* and its bioactive ingredients to inhibit the occurrence and development of CRC.

Combined with the results of network pharmacology, molecular docking, and experimental verification of cell and animal and human tissue samples at three levels, *PM* promotes colorectal cell apoptosis and inhibits the development of CRC mainly by inhibiting the expression of *RelA*. Among the five bioactive ingredients associated with CRC in *PM*, kaempferol and *RelA* had the highest molecular docking level. We also verified the effect of kaempferol on *RelA* and CRC cells. However, there are also limitations. First, *PM* is a natural botanical medicine containing many components, so pharmaceutical composition identification should be conducted. Second, other bioactive ingredients of *PM* should also be verified by experiments. Finally, it is necessary to further explore the direct regulation of bioactive ingredients on *RelA*.

## Data Availability

The datasets presented in this study can be found in online repositories. The names of the repository/repositories and accession number(s) can be found below: https://www.ncbi.nlm.nih.gov/, GSE110225; https://www.ncbi.nlm.nih.gov/, GSE156355; https://www.ncbi.nlm.nih.gov/, GSE164191.

## Abbreviations

TCM, Chinese traditional medicine; CRC, colorectal cancer; *PM*, *Prunus mume*; TCMSP, TCM Systematic Pharmacology Database; GO, biological functions of gene ontology; KEGG, Kyoto Encyclopedia of Genes and Genomes; CHM, Chinese herbal medicine; MF, mumefural; IQ, isoquercitrin; IKK, the upstream IκB kinase; RHD, Rel homologous domain.
