# Supplementary material for: Network Pharmacology and Molecular Docking–Based Investigation: Prunus mume Against Colorectal Cancer via Silencing RelA Expression
Source: Front Pharmacol. 2021 Nov 19;12:761980. doi: 10.3389/fphar.2021.761980 (PMC8640358; doi:10.3389/fphar.2021.761980)
Supplement: Supplementary file 1 [file DataSheet1.docx]

**
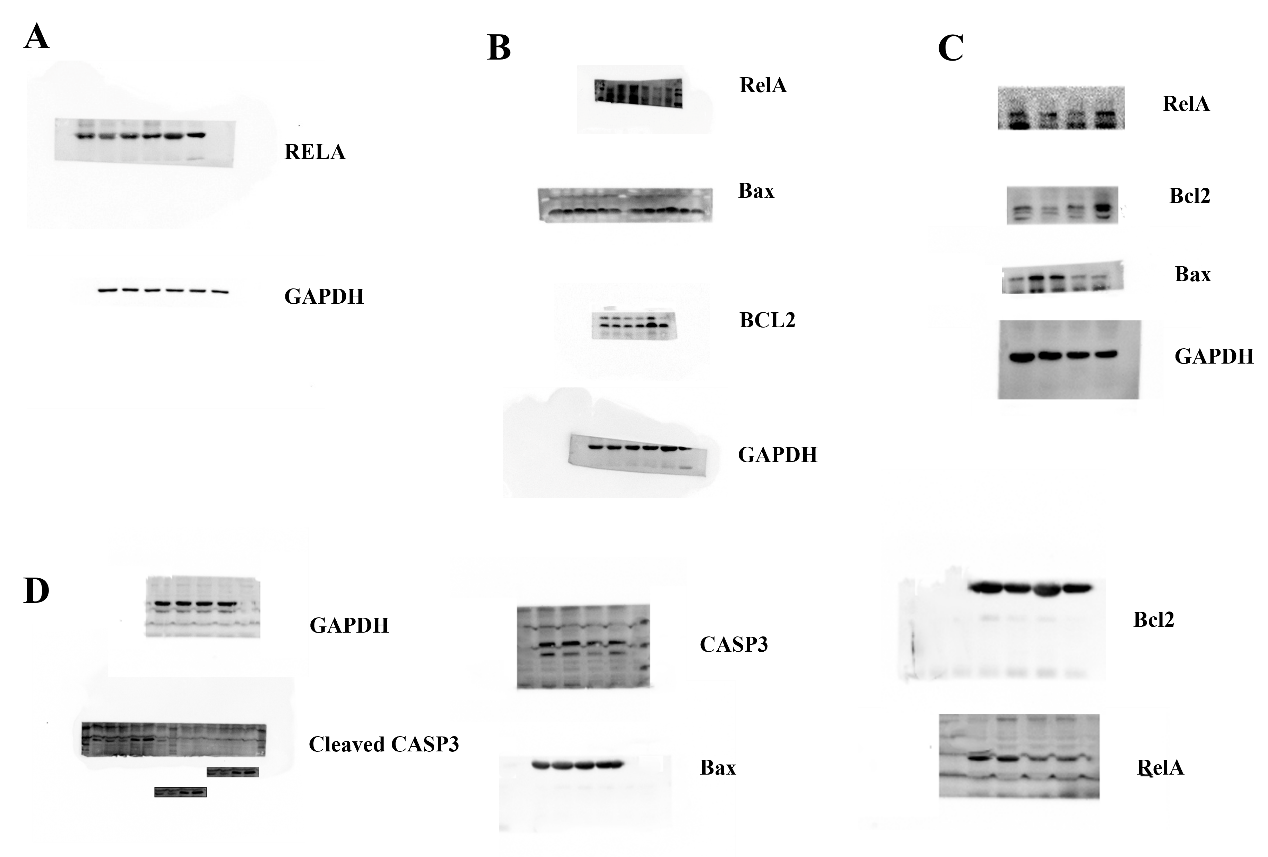
**

Supplemental figure 1**. The original western blots of experimental section.**

**(A)**  Western blot resuilts of RelA in human colon tissue samples. (**B**) Western blot assay verified the expression of Bax, Bcl2 and RELA in HCT116 cells at the concentration of 60 uM,90 uM and 120 uM, respectively. (**C**) After overexpressed RELA plasmid and no-loaded plasmid were transfected, Western blot resuilts in HCT116 cell lines. (**D**) Western blot assays confirmed that FM inhibition of colon cancer in mice was mediated by apoptosis-related pathways.


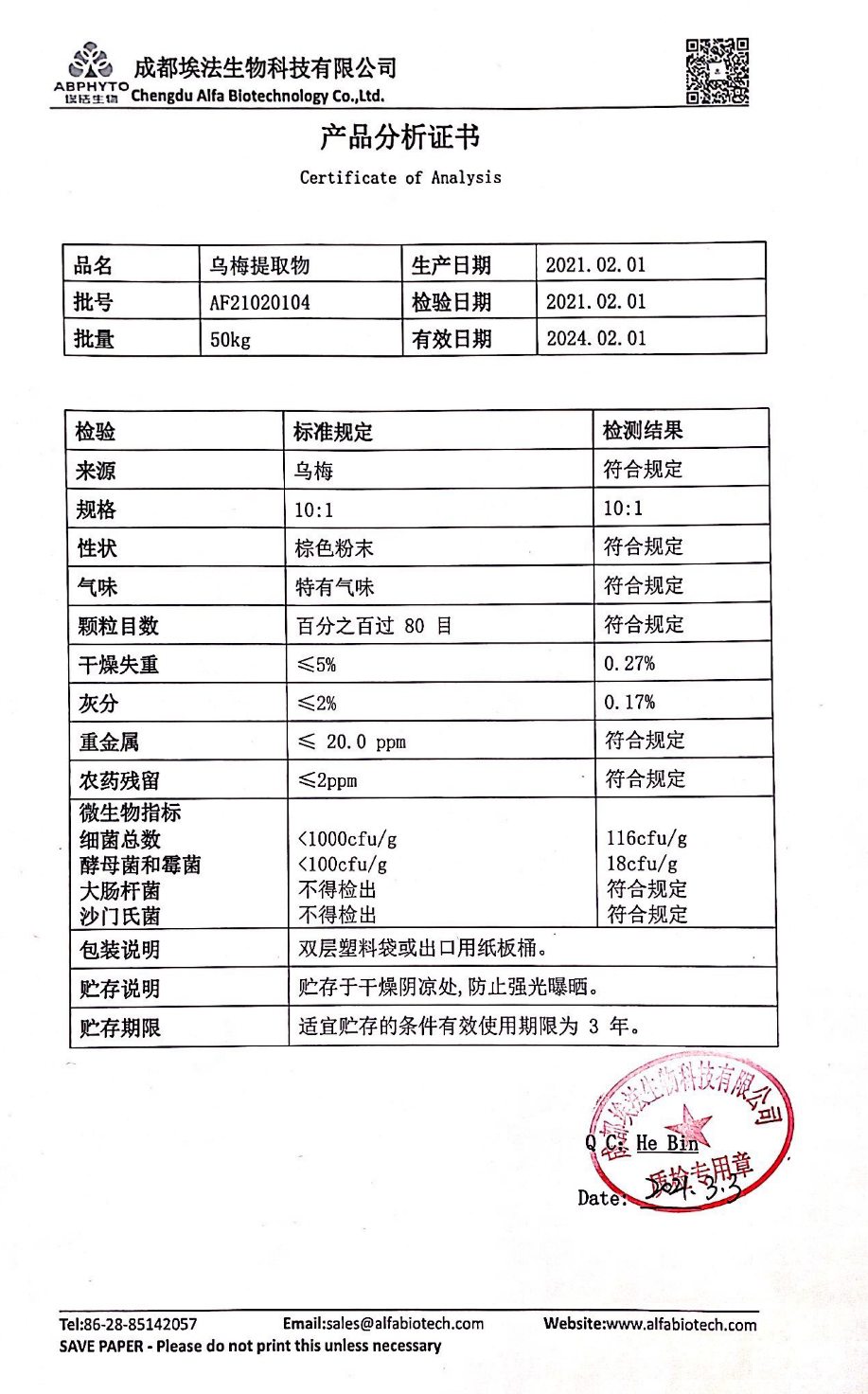


Supplemental figure 2. **Certificate of analysis in Wumei**

Plant extract takes plants as raw materials, and according to the needs of the final product extracted, it is obtained and concentrated through the physical and chemical extraction and separation process. A product formed by one or more of the active ingredients in a substance without changing the structure of the active ingredients. Wumei is extracted on a proportion, which is the powder made after the extraction and concentration of plants raw materials. The means of proportion is the mathematical proportion of the amount of raw materials before extraction and the product after extraction and concentration. Proportional extracts generally do not have a very specific composition and content.Wumei was extracted from 10 kg of raw material and condensed into 1 kg of powder product.
